# Association between intrinsic capacity, changes in intrinsic capacity, and cardiometabolic multimorbidity: results from three prospective cohort studies

**DOI:** 10.3389/fendo.2025.1738997

**Published:** 2025-12-17

**Authors:** Ziyue Wang, Lu Wang, Lulu Tang, Xiaorui Li, Yu Lu, Xiaoli Yu, Fei Liu, Xiaoping Zhu

**Affiliations:** 1 Department of Nursing, Shanghai Tenth People’s Hospital, Tongji University School of Medicine, Shanghai, China; 2 School of Nursing, Anhui University of Chinese Medicine, Hefei, China

**Keywords:** aging, cardiometabolic multimorbidity, dynamic changes, intrinsic capacity, prospective cohort study

## Abstract

**Background:**

Intrinsic capacity (IC)is closely associated with cardiometabolic health in middle-aged and older adults. The purpose of this study was to determine the associations of baseline IC, cumulative IC scores, and their dynamic changes with the risk of incident cardiometabolic multimorbidity (CMM).

**Methods:**

Using data from three prospective cohorts, the China Health and Retirement Longitudinal Study (CHARLS), the English Longitudinal Study of Ageing (ELSA), and the Health and Retirement Study (HRS), participants who met the eligibility criteria were included in this study. Kaplan-Meier curves and Cox models analyzed risk trends and associations.

**Results:**

A total of 11,916 participants were included based on the inclusion and exclusion criteria. At baseline, the risk of CMM in the injured group was significantly higher than that in the non-injured group(Pooled: HR = 1.40, 95% CI 1.30-1.52, *P* < 0.001); Cumulative IC scores showed a graded association with CMM risk: individuals with moderately reduced scores (≈1 SD below intact) had higher risk (HR = 1.22, 95% CI 1.09–1.36), and those with substantially reduced scores (>1 SD below intact) had even higher risk (HR = 1.61, 95% CI 1.47–1.78). For dynamic changes, CMM risk was significantly higher in persistent impairment and decline groups; even the improvement group had higher risk than the no-decline group (Decline: HR = 1.25, 95% CI 1.10-1.42, *P* < 0.001; Improvement: HR = 1.28, 95% CI 1.13-1.45, *P* < 0.001; Persistent impairment: HR = 1.63, 95% CI 1.48-1.80, *P* < 0.001).

**Conclusions:**

IC and its changes relate to CMM risk. Even with IC improvement, risk remains higher than in those with intact IC. Precise strategies to delay IC decline and individualized interventions are needed for CMM control.

## Introduction

Cardiometabolic diseases (CMD) are a group of diseases related to cardiac function and metabolic processes, with common types including hypertension, diabetes mellitus, coronary heart disease, and stroke. When an individual has two or more of these diseases concurrently, it is defined as cardiometabolic multimorbidity (CMM) (1). Evidence suggests that CMM is associated with a variety of adverse health outcomes; compared with individuals without diseases, the life expectancy of patients with CMM can be shortened by 15 years (2, 3). In recent years, with the annual increase in the prevalence and incidence of CMM, CMM triggered by the decline in individuals’ physical capacity and mental health has gradually attracted significant attention from experts (4–6).

In 2015, the World Health Organization (WHO) officially proposed a new public health action framework (7). This framework innovatively introduced the concept of intrinsic capacity (IC), which is defined as a core indicator for comprehensively assessing an individual’s physical and mental capacities, and mainly includes locomotion, vitality, cognition, psychological, and sensory as its five dimensions (8). Data from a large-scale study based on the UK Biobank demonstrates that participants with low IC levels at baseline were significantly associated with subsequent disease-specific mortality (hypertension, stroke, and coronary heart disease), suggesting that IC may be a key predictive indicator for the prognosis of these diseases (5). From a behavioral perspective, the decline in IC may lead to changes in lifestyle, such as reduced physical activity or increased social isolation, both of which can further increase the risk of CMM (9).

Currently, evidence regarding the association between IC and adverse health outcomes has also been accumulating. However, several research gaps remain. First, most study outcomes focus on the association between IC and a single CMD, with no targeted exploration of CMM as a distinct form of multimorbidity. This fails to reflect the true characteristics of multimorbidity in this population, limiting clinical applicability (4). Second, study designs are mainly based on single IC measurement at baseline or cross-sectional analyses, failing to capture the dynamic trajectories of IC. This limitation prevents clarification of the association between changes in IC and the risk of CMM, thus hindering the development of early warning protocols (10). Third, study populations are mostly limited to single national cohorts, lacking cross-country validation, which leads to restricted external validity and further limits its standardized application in the prevention and control of CMM among the global population aged ≥50 years.

Based on the discussed context, this study aims to investigate the association between IC, its changes, and CMM. The analysis was performed using three prospective databases: the China Health and Retirement Longitudinal Study (CHARLS), the English Longitudinal Study of Ageing (ELSA), and the Health and Retirement Study (HRS). This study enhances the generalizability of results through cross-national cohorts; meanwhile, it can explore the heterogeneity of the associations between IC, its changes, and CMM among populations in different regions. The study will overcome the limitation of sole baseline measurement in a single cohort and combine it with the Cox proportional hazards model to analyze the impact of different IC levels and trajectories on incident CMM. Theoretically, this study can fill the research gap in the association between dynamic IC trajectories and CMM in populations aged ≥50 years, while verifying the necessity of multi-cohort analysis in geriatric cardiometabolic health research. These methods can inform the development of future intervention strategies for CMM.

## Methods

### Study design and participants

CHARLS, ELSA, and HRS are nationally representative prospective cohort studies conducted in China, the United Kingdom, and the United States, respectively (11–13). In this study, Wave 1 (2011) of CHARLS, Wave 2 (2004-05) of ELSA, and Wave 10 (2010) of HRS were defined as the baselines. Wave 2 (2013) of CHARLS, Wave 4 (2008-09) of ELSA, and Wave 11 (2012) of HRS were regarded as the second surveys. This study used data from the baselines and the second surveys to assess the status and dynamic changes of IC. Subsequent follow-up surveys were used to track the outcome until the final follow-up surveys which were Wave 5 (2020) of CHARLS, Wave 9 (2018-19) of ELSA, and Wave 15 (2020) of HRS. CHARLS, ELSA, and HRS have obtained approvals from the Ethics Review Boards of Peking University, the London Multi-Center Study, and the University of Michigan, respectively. All participants provided informed consent forms. The authors did not participate in direct data collection, and the ownership of the data remains with the original data custodians.

**Figure 1:**
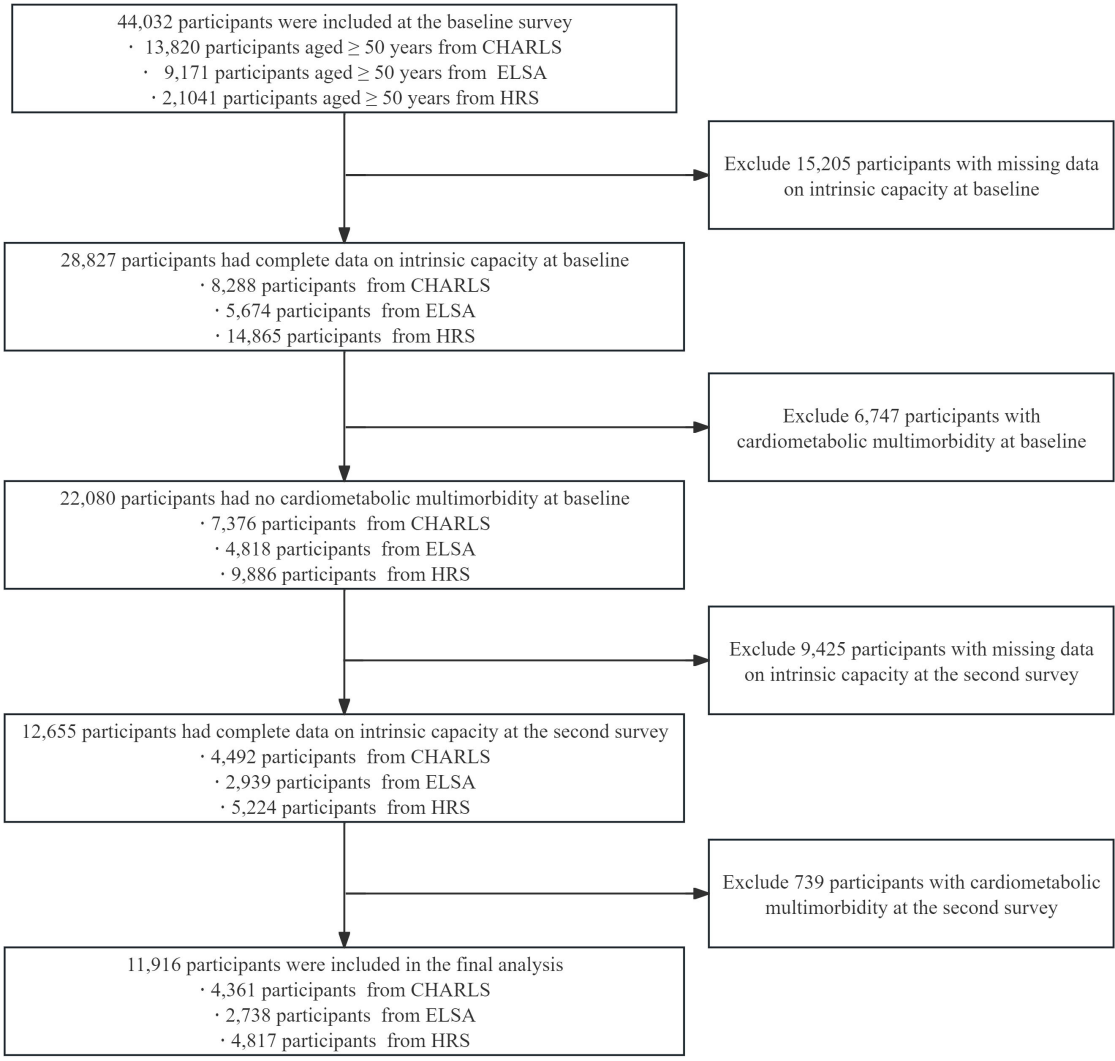
Selection process of the study population.

**Figure 1** presents the selection process of the study population. A total of 44,032 participants aged over 50 years from CHARLS, ELSA, and HRS were included in the screening. First, this study excluded 15,205 participants with missing IC data at baseline. Second, 6,747 participants who had already been diagnosed with CMM at baseline were excluded. To explore the dynamic changes of IC, 9,425 participants with missing IC data in the second survey were further excluded. Finally, 739 participants who had CMM in the second survey were excluded. Ultimately, 11,916 participants were included in the final analysis.

### IC assessment

IC was assessed following the World Health Organization’s Integrated Care for Older People (ICOPE) screening tool, which evaluates an individual’s physical and mental reserve across five domains—cognition, psychological, sensory(vision and hearing), vitality, and locomotion. This operational approach has been widely applied and validated in population-based aging studies (14–17). To ensure comparability between CHARLS, ELSA, and HRS, each domain was modeled using tunable variables from each cohort. Each field is encoded as 0 (damaged) or 1 (complete). The IC score ranges from 0 to 6 points. A higher score indicates a better IC level—with 6 points indicating fully intact IC and a score of ≤5 points indicating impaired IC (18–20). Although IC consists of five domains, the sensory domain includes two separate indicators (vision and hearing), resulting in six items in total and therefore a total score of 0 to 6.

To further characterize IC status across time, two IC-related indicators were derived using data from the baseline and the second follow-up assessments: cumulative IC scores and changes in IC. Cumulative IC scores represent the overall IC level across the two waves and were calculated as the sum of the IC scores at baseline and follow-up, using the formula:


$$Cumulative\;IC\;scores=IC_{baseline}+IC_{follow-up}$$

Based on its empirical distribution, cumulative IC scores were categorized into three levels (T1–T3), where T1 represents intact cumulative IC, T2 represents cumulative IC scores approximately one standard deviation below the intact level, and T3 indicates cumulative IC scores more than one standard deviation below the intact level (distribution shown in **Supplementary Figure S1**).

Changes in IC were evaluated both continuously and categorically. The continuous change score was defined as:


$$changes\;in\;IC=IC_{follow-up}-IC_{baseline}$$

For categorical analyses, participants were classified into four mutually exclusive groups reflecting their IC trajectories: no decline (intact IC at both assessments), decline (intact at baseline but impaired at follow-up), improvement (impaired at baseline but intact at follow-up), and persistent impairment (impaired at both assessments).

Details of the domain construction and variable harmonization are provided in **Supplementary Table S1**.

### Covariates assessment

In the analytical framework of this study, the following covariates were included: age, gender, educational level, marital status, smoking status, drinking status, systolic blood pressure (SBP), diastolic blood pressure (DBP), and handgrip strength. In order to ensure data consistency across the three databases (CHARLS, ELSA, HRS), this study standardized definitions for covariates. Educational level was uniformly categorized into three groups: below high school, high school, and above high school. Marital status was divided into two categories: the married group, which included married individuals and those who were married but had absent spouses, and the other group, which encompassed separated, divorced, widowed, and never-married individuals. Smoking status was classified into two categories: past or current smokers and never smokers. Drinking status was similarly divided into two groups: past or current drinkers and never drinkers.

### Outcome definition and follow-up

The primary outcome of this study was incident CMM. Across CHARLS, ELSA, and HRS, CMM was defined based on participants’ self-reported doctor-diagnosed conditions, including hypertension, diabetes or elevated blood glucose, heart disease (e.g., myocardial infarction, coronary heart disease, angina pectoris, congestive heart failure, and other heart diseases), and stroke. Coexistence of 2 or more of these cardiometabolic diseases was considered as CMM.

During the follow-up period, the follow-up endpoints were the first occurrence of CMM, death, and the observation cutoff date, whichever came first. Death data in CHARLS and HRS were obtained from the most recent survey, while death data in ELSA were only available up to Wave 6 (2012-13). The observation cutoff date was the date of the last survey that participants attended; ideally, this corresponded to Wave 5 (2020) of CHARLS, Wave 9 (2018-19) of ELSA, and Wave 15 (2020) of HRS.

### Statistical analysis

In descriptive statistics, continuous variables were expressed as mean [standard deviation (SD)], and categorical variables were presented as counts (percentages).

Pooled associations across CHARLS, ELSA, and HRS were estimated using a one-stage stratified Cox proportional hazards model, with cohort specified as a stratification variable to allow cohort-specific baseline hazards.

To analyze the association between baseline IC and the risk of incident CMM, Cox proportional hazards regression models were used to calculate the hazard ratio (HR) and its 95% confidence interval (95% CI). With the intact IC group as the reference group, three regression models were fitted: Model 1: Unadjusted model; Model 2: Adjusted for demographic characteristics including age, gender, educational level, and marital status; Model 3: Further adjusted for smoking status, drinking status, systolic blood pressure (SBP), diastolic blood pressure (DBP), and handgrip strength on the basis of Model 2. Missing values of covariates were handled using multiple imputation. In addition to baseline IC, the associations of cumulative IC scores and changes in IC with incident CMM were also examined. Cumulative IC scores were categorized into three levels (T1–T3) based on their empirical distribution, and changes in IC were classified into four trajectory groups (no decline, decline, improvement, and persistent impairment), as described previously. All the above analyses were adjusted for covariates, and the proportional hazards assumption of each Cox regression model was verified using the Schoenfeld residual test (21).

To verify the robustness of the results, multiple sensitivity analyses were conducted in this study: (I) To reduce potential bias introduced by multiple imputation, after excluding samples with missing covariates, the associations between baseline IC score, cumulative IC scores, changes in IC, and the risk of incident CMM were re-analyzed; (II) To reduce potential residual confounding bias, a supplementary analysis was performed using the propensity score matching (PSM) method; (III) To treat death as a competing event for CMM, we used a competing risks model to re-verify the association effects. (IV) To address potential heterogeneity within the improvement group, we optimized the behavior of IC changes in sensitivity analysis. Based on the transition pattern from baseline to follow-up, IC changes were divided into five mutually exclusive categories: No decline (The IC is intact both at baseline and the second survey), Decline (The IC is intact at baseline but impaired at the second survey), Minor improvement (Baseline IC shows mild impairment (IC = 5), and becomes intact at the second survey), Major improvement (Baseline IC shows substantial impairment (IC< 5), and becomes intact at the second survey), Persistent impairment (The IC is impaired both at baseline and the second survey). (V) To address potential confounding caused by baseline heterogeneity in pre-existing single cardiometabolic conditions (e.g., hypertension, diabetes, heart disease, or stroke), we conducted an additional sensitivity analysis stratified by single-disease subgroups.

## Results

### Characteristics of the study population

According to the inclusion and exclusion criteria, a total of 11,916 participants (females: 54.6%, mean age: 66.7 years) were included from CHARLS, ELSA, and HRS for the baseline IC characteristic analysis, with specific breakdowns as follows: 4,361 participants from CHARLS (females: 43.5%, mean age: 60.8 years), 2,738 participants from ELSA (females: 60.8%, mean age: 63.1 years), and 4,817 participants from HRS (females: 61.1%, mean age: 74.0 years). Detailed baseline characteristics of these participants are presented in **Table 1**. Across CHARLS, ELSA, and HRS, a higher proportion of participants had an educational level of high school or below. Specifically, this proportion reached 98.8% in CHARLS, which was much higher than the 81.3% in ELSA and 76.3% in HRS. Regarding marital status, CHARLS had the highest proportion of married participants (88.9%), while HRS had the lowest (59.6%). Compared with non-drinkers, the proportion of drinkers was extremely high in ELSA (93.4%), whereas the lowest proportion of drinkers was observed in CHARLS (46.6%). For smoking status, 58.3% of participants in ELSA were smokers, which was higher than the proportions in CHARLS (46.3%) and HRS (54.7%). In terms of health indicators, compared with the other cohorts, HRS had the highest mean DBP (78.5 mmHg) and the lowest mean grip strength (30.0 kg). Details of baseline characteristics of IC status for CHARLS, ELSA, and HRS participants are presented in **Supplementary Tables S2**–**S4**.

**Table 1:** Baseline characteristics of participants in CHARLS, ELSA, and HRS.

Variables	Overall	CHARLS	ELSA	HRS
Number	11916	4361	2738	4817
Age, mean (SD), years	66.7 (9.5)	60.8 (7.2)	63.1 (7.9)	74.0 (6.9)
Sex, n (%)				
Female	6509 (54.6)	1899 (43.5)	1666 (60.8)	2944 (61.1)
Male	5407 (45.4)	2462 (56.5)	1072 (39.2)	1873 (38.9)
Education, *n* (%)				
Below high school	5526 (47.4)	3877 (88.9)	771 (30.9)	878 (18.2)
High school	4484 (38.4)	430 (9.9)	1256 (50.4)	2798 (58.1)
College or above	1660 (14.2)	54 (1.2)	467 (18.7)	1139 (23.7)
Marital status, *n* (%)				
Married or partnered	8686 (72.9)	3879 (88.9)	1938 (70.8)	2869 (59.6)
Other marital status	3230 (27.1)	482 (11.1)	800 (29.2)	1948 (40.4)
Drinking status, *n* (%)				
Never drinkers	4642 (39.5)	2329 (53.4)	171 (6.6)	2142 (44.5)
Ever drinkers	7106 (60.5)	2030 (46.6)	2401 (93.4)	2675 (55.5)
Smoking status, *n* (%)				
Never smokers	5648 (47.6)	2344 (53.7)	1140 (41.7)	2164 (45.3)
Ever smokers	6228 (52.4)	2017 (46.3)	1595 (58.3)	2616 (54.7)
SBP, mean (SD), mmHg	131.2 (19.6)	129.6 (20.2)	132.5 (17.6)	132.9 (20.4)
DBP, mean (SD), mmHg	76.1 (11.3)	75.3 (11.7)	75.7 (10.4)	78.5 (11.1)
Grip strength, mean (SD), kg	32.4 (10.5)	33.8 (9.9)	32.1 (11.2)	30.0 (10.5)

### Baseline IC and its association with CMM

To assess the association between baseline IC and CMM, this study utilized Kaplan-Meier survival curves stratified by baseline IC score. Results showed that participants with impaired baseline IC had a higher risk of developing CMM than those with intact baseline IC (**Supplementary Figure S2**). Furthermore, this study further adjusted Cox proportional hazards models to explore the association between baseline IC and CMM in depth (**Table 2**). Results indicated that, with the intact baseline IC group as the reference, participants with impaired baseline IC had a significantly increased risk of CMM in all individual cohorts and the pooled population (CHARLS: HR = 1.36, 95% CI: 1.17-1.52, *P* < 0.001; ELSA: HR = 1.51, 95% CI: 1.25-1.82, *P* < 0.001; HRS = 1.35, 95% CI: 1.20-1.53, *P* < 0.001; Pooled: HR = 1.40, 95% CI: 1.30-1.52, *P* < 0.001). This association remained stable even after adjusting for confounding factors.

**Table 2:** Association between baseline IC and CMM.

Model	CHARLS		ELSA		HRS		Pooled	
	HR (95% CI)	*P*	HR (95% CI)	*P*	HR (95% CI)	*P*	HR (95% CI)	*P*
Model1	1.31 (1.15-1.48)	<0.001	1.69 (1.41-2.03)	<0.001	1.35 (1.20-1.52)	<0.001	1.51 (1.39-1.63)	<0.001
Model2	1.26 (1.10-1.43)	<0.001	1.51 (1.25-1.83)	<0.001	1.34 (1.19-1.52)	<0.001	1.38 (1.27-1.50)	<0.001
Model3	1.36 (1.17-1.52)	<0.001	1.51 (1.25-1.82)	<0.001	1.35 (1.20-1.53)	<0.001	1.40 (1.30-1.52)	<0.001

*CHARLS* China Health and Retirement Longitudinal Study, *ELSA* English Longitudinal Study of Ageing, *HRS* Health and Retirement Study. Model 1 was the unadjusted model (no covariates adjusted). Model 2 was adjusted for age, sex, education level, and marital status. Model 3 was further adjusted for smoking status, drinking status, SBP, DBP, and grip strength, based on Model 2.

### Cumulative IC scores and its association with CMM

**Table 3** presents the association between cumulative IC scores and CMM. In the CHARLS cohort, compared with the T1 group, the CMM risk was significantly higher in both the T2 group (HR = 1.33, 95% CI: 1.12–1.57, *P* = 0.001) and the T3 group (HR = 1.60, 95% CI: 1.37–1.88, *P* < 0.001). In the ELSA cohort, the CMM risk was also significantly elevated in the T2 group (HR = 1.36, 95% CI: 1.07–1.72, *P* = 0.009) and the T3 group (HR = 2.15, 95% CI: 1.73–2.66, *P* < 0.001), with the T3 group showing the largest risk increase among all cohorts. In the HRS cohort, only the T3 group exhibited a significantly higher CMM risk than the T1 group (HR = 1.43, 95% CI: 1.24–1.65, *P* < 0.001). Analysis of the pooled population revealed that the CMM risk was significantly increased in both the T2 group (HR = 1.22, 95% CI: 1.09–1.36, *P* < 0.001) and the T3 group (HR = 1.61, 95% CI: 1.47–1.78, *P* < 0.001). Additionally, the Kaplan-Meier survival curve based on cumulative IC scores classification in this study showed an overall trend that the lower the cumulative IC scores were, the higher the CMM risk was. (**Supplementary Figure S3**).

**Table 3:** Association between cumulative IC scores and CMM.

IC level	CHARLS		ELSA		HRS		Pooled	
	HR (95% CI)	*P*	HR (95% CI)	*P*	HR (95% CI)	*P*	HR (95% CI)	*P*
T1	1 Ref.		1 Ref.		1 Ref.		1 Ref.	
T2	1.33 (1.12-1.57)	0.001	1.36 (1.07-1.72)	0.009	1.08 (0.91-1.28)	0.374	1.22 (1.09-1.36)	<0.001
T3	1.60 (1.37-1.88)	<0.001	2.15 (1.73-2.66)	<0.001	1.43 (1.24-1.65)	<0.001	1.61 (1.47-1.78)	<0.001

The cumulative IC scores are calculated by summing the IC score at baseline and that at the second survey. On this basis, three grades are classified: T1 indicates the cumulative IC scores are intact; T2 indicates the cumulative IC scores are 1 standard deviation below the intact level; T3 indicates the cumulative IC scores are 1 standard deviation below the intact level more than 1 standard deviation below the intact level.

*CHARLS* China Health and Retirement Longitudinal Study, *ELSA* English Longitudinal Study of Ageing, *HRS* Health and Retirement Study.

All models were adjusted for age, sex, education level, marital status, smoking status, drinking status, SBP, DBP, and grip strength.

### Association between changes in IC and CMM

**Table 4** presents the association between changes in IC and CMM. Results showed that, with the group without IC decline as the reference, the group with persistent impairment had a significantly increased risk of CMM across all cohorts (CHARLS: HR = 1.65, 95% CI: 1.40–1.95, *P* < 0.001; ELSA: HR = 2.26, 95% CI: 1.79–2.79, *P* < 0.001; HRS: HR = 1.41, 95% CI: 1.22–1.63, *P* < 0.001; Pooled: HR = 1.63, 95% CI: 1.48–1.80, *P* < 0.001). The group with IC decline also exhibited a significantly elevated risk of CMM(CHARLS: HR = 1.41, 95% CI: 1.17–1.71, *P* < 0.001; ELSA: HR = 1.44, 95% CI: 1.08–1.92, *P* = 0.012; HRS: HR = 1.04, 95% CI:0.85–1.28, *P* = 0.689; Pooled: HR = 1.25, 95% CI: 1.10–1.42, *P* < 0.001). Notably, the group with IC improvement also had a significantly higher risk of CMM (CHARLS: HR = 1.28, 95% CI: 1.05–1.56, *P* = 0.015; ELSA: HR = 1.37, 95% CI: 1.04–1.79, *P* = 0.023; HRS: HR = 1.24, 95% CI: 1.02–1.51, *P* = 0.032; Pooled: HR = 1.28, 95% CI: 1.13–1.45, *P* < 0.001). Additionally, Kaplan-Meier survival curves stratified by changes in IC, used in this study, are provided in **Supplementary Figure S4**.

**Table 4:** Association between changes in IC and CMM.

IC trajectory	CHARLS		ELSA		HRS		Pooled	
	HR (95% CI)	*P*	HR (95% CI)	*P*	HR(95% CI)	*P*	HR(95% CI)	*P*
No decline	1 Ref.		1 Ref.		1 Ref.		1 Ref.	
Decline	1.41 (1.17-1.71)	<0.001	1.44 (1.08-1.92)	0.012	1.04 (0.85-1.28)	0.689	1.25 (1.10-1.42)	<0.001
Improvement	1.28 (1.05-1.56)	0.015	1.37 (1.04-1.79)	0.023	1.24 (1.02-1.51)	0.032	1.28 (1.13-1.45)	<0.001
Persistent impairment	1.65 (1.40-1.95)	<0.001	2.26 (1.79-2.79)	<0.001	1.41 (1.22-1.63)	<0.001	1.63 (1.48-1.80)	<0.001

The changes in IC is calculated by subtracting the IC score at baseline from that at the second survey. On this basis, four grades are classified, No decline: The IC is intact both at baseline and the second survey; Decline: The IC is intact at baseline but impaired at the second survey; Improvement: The IC is impaired at baseline but intact at the second survey; Persistent impairment: The IC is impaired both at baseline and the second survey.

*CHARLS* China Health and Retirement Longitudinal Study, *ELSA* English Longitudinal Study of Ageing, *HRS* Health and Retirement Study. All models were adjusted for age, sex, education level, marital status, smoking status, drinking status, SBP, DBP, and grip strength.

### Sensitivity analyses

(I) To reduce potential bias introduced by multiple imputation, after excluding samples with missing covariates, the associations between baseline IC score, cumulative IC scores, changes in IC, and the risk of incident CMM were re-analyzed. The results were consistent with those of the primary analysis (**Supplementary Tables S5**–**S7**). (II) To mitigate potential residual confounding bias, a supplementary analysis was conducted using the propensity score matching (PSM) method (**Supplementary Tables S8**-**S10**). (III) With death treated as a competing event for CMM, a competing risks model was used to re-verify the association effects (**Supplementary Tables S11**–**S13**). (IV) To further refine the improvement group, we re-analyzed the association between changes in IC and risk of CMM (**Supplementary Table S14**). (V) To minimize potential confounding introduced by baseline heterogeneity in pre-existing single cardiometabolic conditions, we performed a subgroup sensitivity analysis restricted to participants with only one condition at baseline. The pooled results were generally consistent with the main findings (**Supplementary Tables S15**–**S17**).

## Discussion

Using three prospective cohorts, we explored associations of baseline IC, cumulative IC scores, and their dynamic changes with incident CMM risk via Kaplan-Meier curves and Cox models. Results showed baseline IC-impaired participants had higher incident CMM risk than intact ones, with this association stable after adjusting for confounders. Cumulative IC scores correlated negatively with CMM risk, most notably in the largest decline group. IC decline and persistent impairment significantly increased CMM risk; even improved IC carried higher risk than the no-decline reference group. These trends were consistent across individual cohorts and the pooled population.

Previous studies have gradually revealed the association between IC and CMD in middle-aged and older populations. A national cohort study found that for each 1-point increase in baseline IC, the risk of cardiovascular disease (CVD) decreased by 8% (22). Another study further confirmed that impaired baseline IC is an independent predictor of incident CVD (5). These findings are highly consistent with the core results of the present study—across the three cohorts CHARLS, ELSA, HRS and the pooled population, we observed that impaired baseline IC significantly increased the risk of CMM. This cross-ethnic and cross-regional consistency further validates the universality of IC as a risk factor for CMM. From the perspective of basic research, IC and CMD may share common physiological pathways, such as chronic inflammation (23, 24). This provides further evidence to support the core conclusion of this study.

IC dynamic characteristics are not isolated but reflect physiological function evolution in middle-aged and older adults. Two prospective studies showed IC changes dynamically during follow-up, with three trajectories in the Chinese population (6, 25). confirming its non-fixed nature and highlighting the significance of exploring its dynamic associations with CMM risk. Our results reveal differential impacts of IC changes on CMM risk: First, those with persistent IC impairment face the highest risk, as prolonged core physiological dysfunction (e.g., metabolic regulation) maintains low reserve, failing to counter risks and accelerating cardiometabolic disease accumulation (26, 27). This suggests that persistent IC impairment is a high-risk signal for CMM and that this population should be prioritized for clinical intervention. Second, IC decline significantly elevates risk, possibly reflecting worsened lifestyles or underlying diseases; timely intervention to halt decline may reduce comorbidity. Most notably, though IC improvement is positive, their risk remains higher than the no-decline reference group, indicating improvement does not eliminate risk, necessitating long-term monitoring to prevent rebound.

Beyond these findings, it is important to recognize that the two indicators used to characterize IC dynamics—changes in IC and cumulative IC scores—capture distinct aspects of functional evolution, which may help explain their different associations with CMM risk. Changes in IC reflect short-term transitions between two assessments and emphasize whether an individual’s functional reserve improves, declines, or remains stable. In contrast, cumulative IC scores represent the overall functional reserve accumulated across the period and reflect long-term physiological robustness. Consequently, an individual who experiences a small decline from a high IC level may still maintain strong cumulative reserve, whereas someone with persistently low IC may exhibit chronically insufficient reserve despite no recent decline. These conceptual differences suggest that cumulative IC scores may be more relevant to chronic cardiometabolic dysregulation, while IC changes primarily capture transient fluctuations that influence risk when sufficiently marked or sustained.

In addition, the negative correlation between cumulative IC scores and comorbidity risk further supports the importance of long-term IC reserves in shaping cardiometabolic vulnerability. Individuals with lower cumulative IC scores—whether persistent impairment or significant decline—consistently exhibit higher CMM risk. This pattern highlights the accumulation of physiological deficits over time and is a key mechanism linking IC deterioration to cardiometabolic deterioration. These findings break the perception that IC only requires a single assessment and clarify the importance of dynamically monitoring IC changes for CMM risk prediction.

This study holds several strengths. First, it is the first study to investigate the association between IC, changes in IC, and CMM. Additionally, this study includes three prospective cohorts with diverse ethnicities; this cross-population design not only avoids the geographical limitation of a single cohort but also significantly enhances the generalizability of the conclusions through the consistency of results across different populations. Furthermore, the study results were consistent across the three cohorts, indicating the generality of the conclusions. Finally, diverse sensitivity analyses were conducted, which also ensured the robustness of the study results.

This study has several limitations. First, consistent with many previous studies (28–30), outcome variables were defined based on participants’ self-reported doctor-diagnosed information, which may introduce bias. Second, differences in follow-up intervals across cohorts may affect the comparability of results: the interval between CHARLS Wave 1 (2011) and Wave 2 (2013) was 2 years, between ELSA Wave 2 (2004–05) and Wave 4 (2008–09) was 4 years, and between HRS Wave 10 (2010) and Wave 11 (2012) was 2 years. The reason for the different interval between baseline and the second follow-up in ELSA was the lack of available BMI data in ELSA Wave 3, which may have caused minor discrepancies. Finally, in ELSA, death data were only available up to Wave 6, while outcome data were tracked until Wave 9. The lack of death data between Wave 7 and Wave 9 may have led to incorrect estimation of follow-up duration.

## Conclusion

Both IC and changes in IC are associated with the risk of CMM. Even with IC improvement, the associated risk remains higher than that in individuals with intact IC. In the future, it is necessary to develop targeted strategies to delay the decline in IC and implement individualized interventions to improve IC, thereby contributing to the prevention and control of CMM.

## Data Availability

Publicly available datasets were analyzed in this study. This data can be found here: https://g2aging.org/home.
