# Supplementary material for: Association between intrinsic capacity, changes in intrinsic capacity, and cardiometabolic multimorbidity: results from three prospective cohort studies
Source: Front Endocrinol (Lausanne). 2025 Dec 17;16:1738997. doi: 10.3389/fendo.2025.1738997 (PMC12753437; doi:10.3389/fendo.2025.1738997)
Supplement: Supplementary file 1 [file DataSheet1.docx]

**
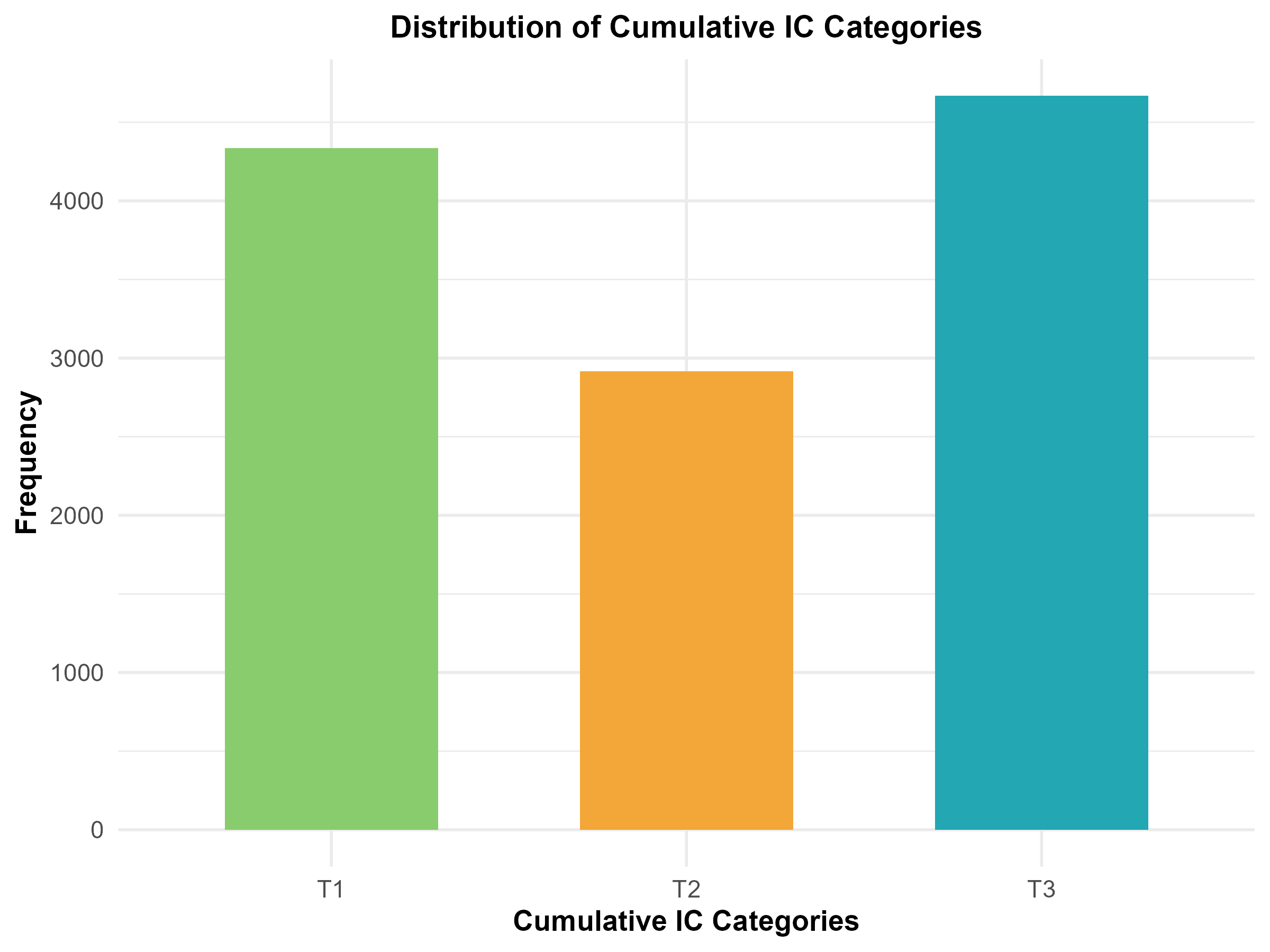
**

**Supplementary Figure 1 Distribution of cumulative IC scores.**

This histogram shows the empirical distribution of cumulative IC scores, which was used to inform the categorization of participants into T1, T2, and T3 groups.


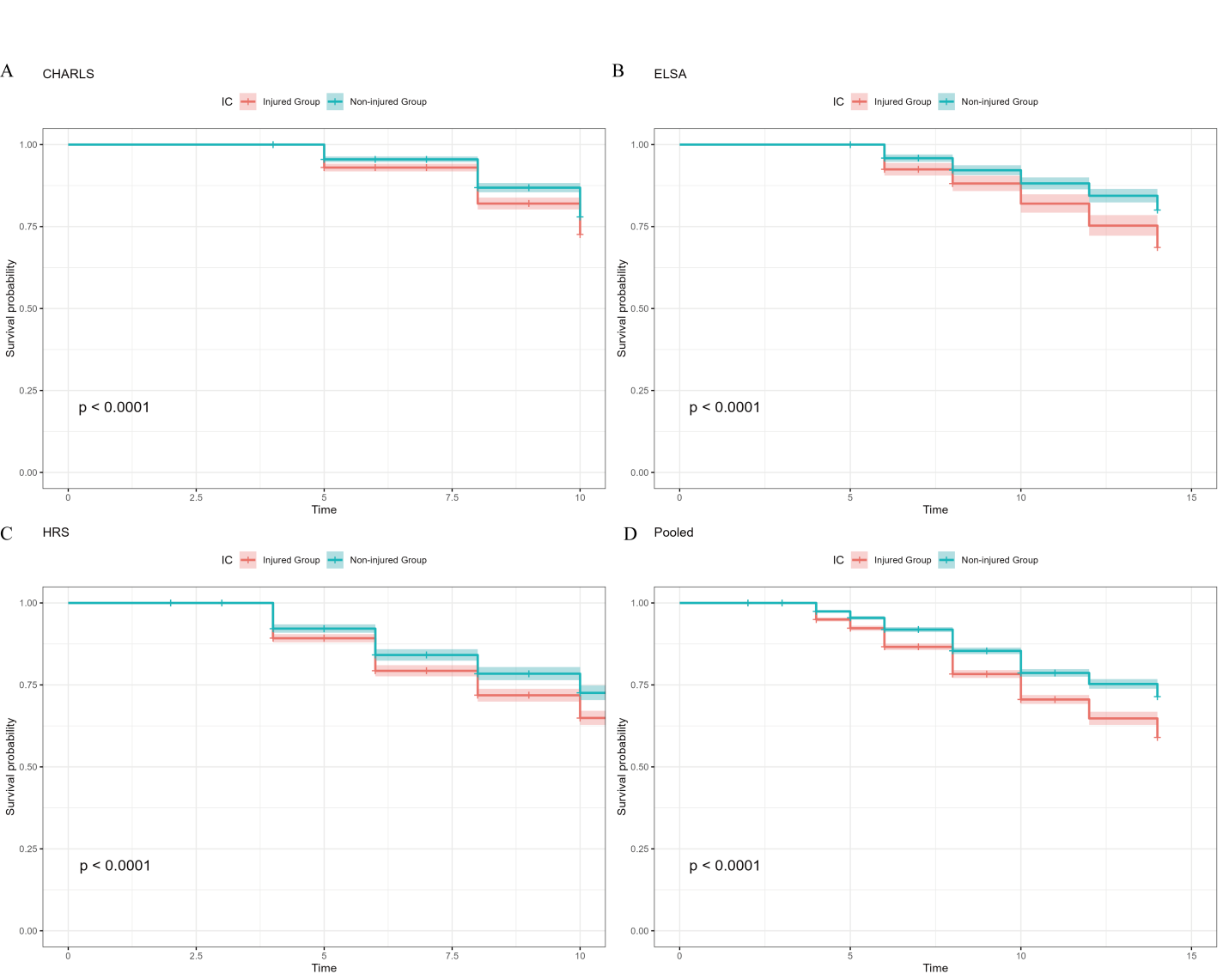


**Supplementary Figure 2 Kaplan-Meier Survival Curve of CMM Based on Baseline IC in CHARLS, ELSA, HRS, and Pooled Analysis**

(A) CHARLS: China Health and Retirement Longitudinal Study; (B) ELSA: English Longitudinal Study of Ageing;(C) HRS: Health and Retirement Study; (D) Pooled.


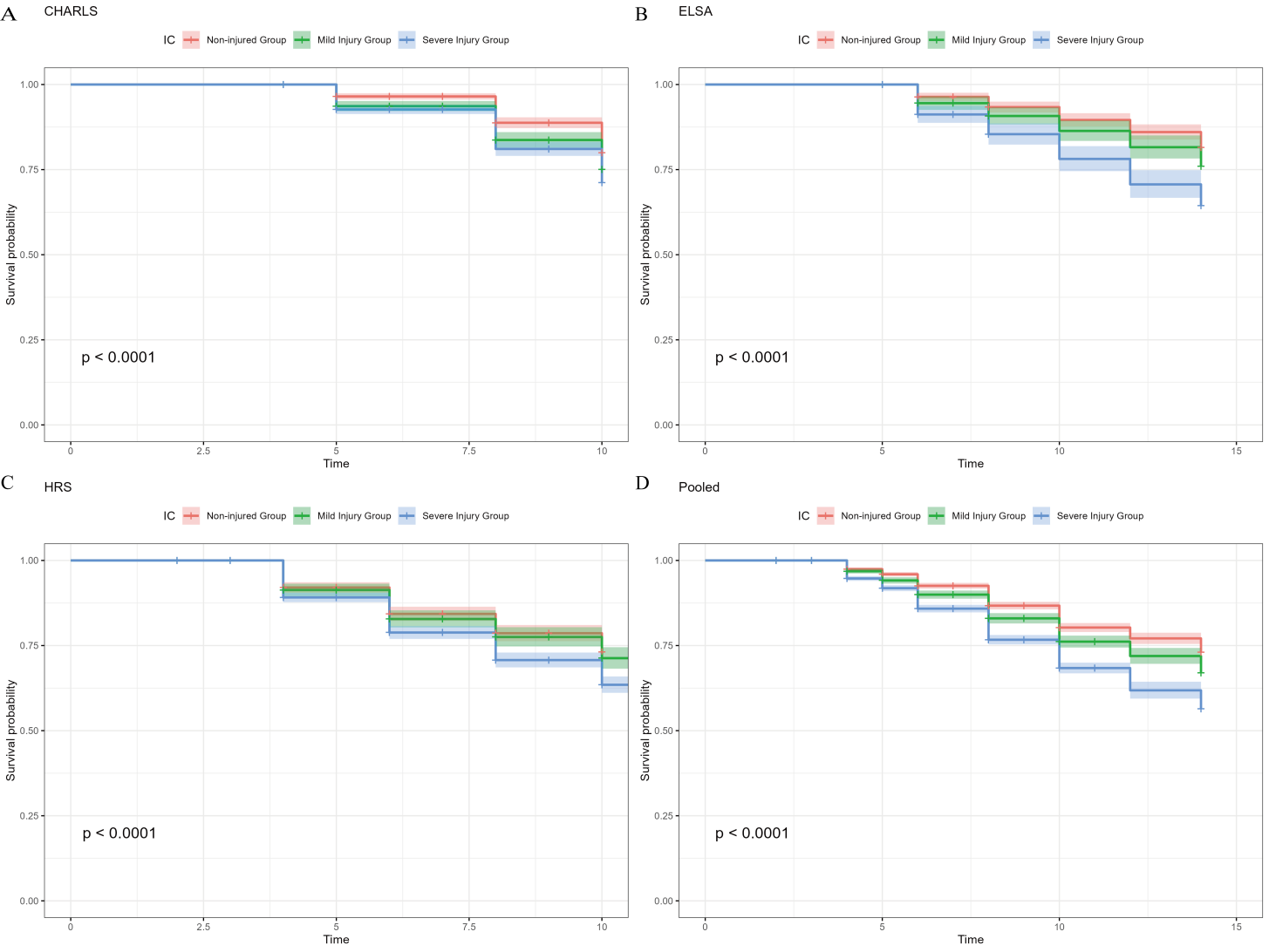


**Supplementary Figure 3 Kaplan-Meier Survival Curve of CMM Based on Cumulative IC scores in CHARLS, ELSA, HRS, and Pooled Analysis**

(A) CHARLS: China Health and Retirement Longitudinal Study; (B) ELSA: English Longitudinal Study of Ageing;(C) HRS: Health and Retirement Study; (D) Pooled.


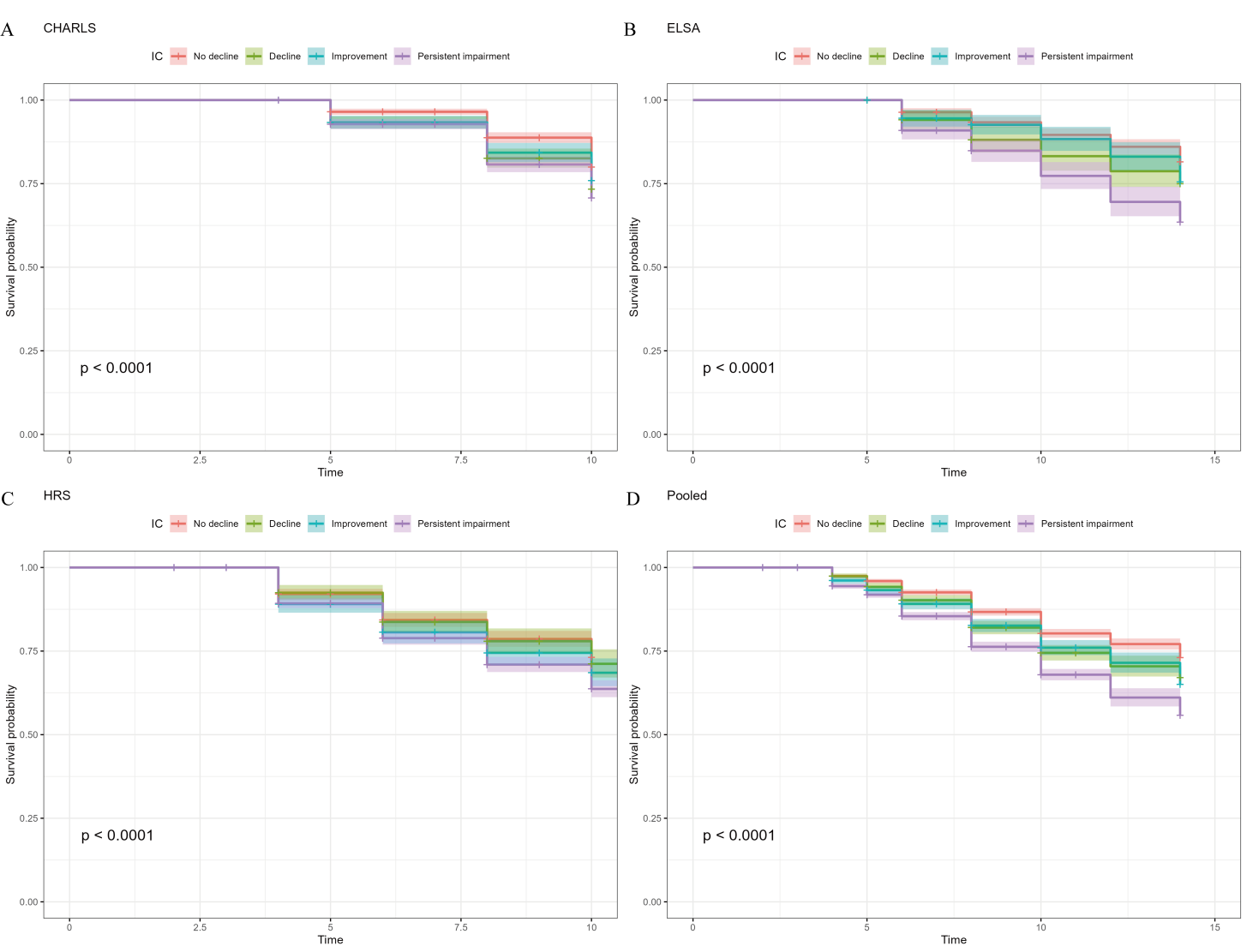


**Supplementary Figure 4 Kaplan-Meier Survival Curve of CMM Based on Changes in IC in CHARLS, ELSA, HRS, and Pooled Analysis**

1. CHARLS: China Health and Retirement Longitudinal Study;(B) ELSA: English Longitudinal Study of Ageing;(C) HRS: Health and Retirement Study; (D) Pooled

**Supplementary Table 1. The description of the fields, subfields and indicators involved in the implementation framework of IC**

| **Domains** | **Measurements** | **CHARLS** | **ELSA** | **HRS** | **Criteria** |
| --- | --- | --- | --- | --- | --- |
| ***Cognition*** | Mini-Mental State Examination, MMSE | Cognition orientation=5 | Cognition orientation=4 | Cognition orientation=4 | Each test score is standardized within the cohort. Participants with a score below the cohort mean >1 SD were classified as impaired (0); Otherwise, it is judged to be complete (1) |
|  |  |  | Fluency in language=0+ | Serial 7’s test=5 |  |
|  |  | Serial 7’s test=5 |  |  |  |
|  |  | Drawing picture=1 | Recall (immediate word recall + delayed word recall)=20 | Recall (immediate word recall + delayed word recall)=20 |  |
|  |  | Recall (immediate word recall + delayed word recall)=20 |  |  |  |
| ***Psychological*** | Center for Epidemiological Studies Depression Scale-10/8 , CESD10/CESD8 | 10 items of core symptoms of depression with following options（CESD10）  Q1: I was bothered by things that don't usually bother me.  Q2: I had trouble keeping my mind on what I was doing.  Q3: I felt depressed.  Q4: I felt everything I did was an effort.  Q5: I felt hopeful about the future.  Q6: I felt fearful.  Q7: My sleep was restless.  Q8: I was happy.  Q9: I felt lonely.  Q10: I could not get "going". | 8 items of core symptoms of depression with following options（CESD8）  Q1: I felt depressed.  Q2: I felt that everything I did was an effort.  Q3: My sleep was restless.  Q4: I was happy.  Q5: I felt lonely.  Q6: I enjoyed life.  Q7: I felt sad.  Q8: I could not get "going". | 8 items of core symptoms of depression with following options（CESD8）  Q1: I felt depressed.  Q2: I felt that everything I did was an effort.  Q3: My sleep was restless.  Q4: I was happy.  Q5: I felt lonely.  Q6: I enjoyed life.  Q7: I felt sad.  Q8: I could not get "going". | If the CES-D score is ≥10 in CHARLS, or ≥3 in ELSA and HRS, it is classified as impaired (0); Otherwise, they are classified as complete (1). |
| ***Sensory*** | Vision | Vision： excellent/very good/ good/fair = 1; poor = 0 | Vision： excellent/very good/ good/fair = 1; poor = 0 | Vision： excellent/very good/ good/fair = 1; poor = 0 | Vision: excellent/very good/good/fair=1; poor=0.  Hearing: excellent/very good/good/fair=1; poor=0.  Total sensory score = Vision + hearing, 0-2 |
|  | Hearing | Hearing: excellent/very good/ good/fair = 1; poor = 0 | Hearing: excellent/very good/ good/fair = 1; poor = 0 | Hearing: excellent/very good/ good/fair = 1; poor = 0 |  |
| ***Vitality*** | BMI | BMI ≤ 18.5 kg/m² =0;  BMI ＞ 18.5 kg/m² =1 | BMI ≤ 18.5 kg/m² =0;  BMI ＞ 18.5 kg/m² =1 | BMI ≤ 18.5 kg/m² =0;  BMI ＞ 18.5 kg/m² =1 | BMI ≤ 18.5 kg/m² =0;  BMI ＞ 18.5 kg/m² =1 |
| ***Locomotion*** | Prioritize Five Independent Sit-to-Stand Tests (5xSTS) If 5xSTS is missing, use Standing up after sitting for a long time | 5xSTS≤14*s=*1, 5xSTS＞14*s=*0/  Standing up after sitting for a long time (No difficulty=1, Have difficulty=0). | 5xSTS≤14*s=*1, 5xSTS＞14*s=*0/  Standing up after sitting for a long time (No difficulty=1, Have difficulty=0). | Standing up after sitting for a long time (Proxy): Due to the unavailability of 5xSTS data in this cohort, only the proxy measure was used. No difficulty=1, Have difficulty=0. | 5xSTS: Completion time≤14*s* scored 1, ＞14*s* scored 0. Proxy: No difficulty scored 1, Have difficulty scored 0. |

**Supplementary Table 2. Baseline Characteristics of the CHARLS Population**

| **Variables** | **Overall** | **Injury Group** | **Non-injury Group** | ***p*** |
| --- | --- | --- | --- | --- |
| Number | 4361 | 1938 | 2423 |  |
| Age, mean (SD), years | 60.8 (7.2) | 61.5 (7.4) | 60.3 (7.0) | <0.01 |
| Sex, n (%) |  |  |  | <0.01 |
| Female | 1899 (43.5) | 997 (51.4) | 902 (37.2) |  |
| Male | 2462 (56.5) | 941 (48.6) | 1521 (62.8) |  |
| Education, n (%) |  |  |  | <0.01 |
| Below high school | 3877 (88.9) | 1804 (93.1) | 2073 (85.6) |  |
| High school | 430 (9.9) | 123 (6.3) | 307 (12.7) |  |
| College or above | 54 (1.2) | 11 (0.6) | 43 (1.8) |  |
| Marital status, n (%) |  |  |  | <0.01 |
| Married or partnered | 3879 (88.9) | 1679 (86.6) | 2200 (90.8) |  |
| Other marital status | 482 (11.1) | 259 (13.4) | 223 (9.2) |  |
| Drinking status, n (%) |  |  |  | <0.01 |
| Never drinkers | 2329 (53.4) | 1114 (57.5) | 1215 (50.2) |  |
| Ever drinkers | 2030 (46.6) | 823 (42.5) | 1207 (49.8) |  |
| Smoking status, n (%) |  |  |  | <0.01 |
| Never smokers | 2344 (53.7) | 1133 (58.5) | 1211 (50.0) |  |
| Ever smokers | 2017 (46.3) | 805 (41.5) | 1212 (50.0) |  |
| SBP, mean (SD), mmHg | 129.6 (20.2) | 128.6 (20.9) | 130.4 (19.6) | <0.01 |
| DBP, mean (SD), mmHg | 75.3 (11.7) | 74.4 (11.9) | 75.9 (11.6) | <0.01 |
| Grip strength, mean (SD), kg | 33.8 (9.9) | 31.4 (9.5) | 35.7 (9.7) | <0.01 |

**Supplementary Table 3. Baseline Characteristics of the ELSA Population**

| **Variables** | **Overall** | **Injury Group** | **Non-injury Group** | ***p*** |
| --- | --- | --- | --- | --- |
| Number | 2738 | 1111 | 1627 |  |
| Age, mean (SD), years | 63.1 (7.9) | 64.5 (8.6) | 62.1 (7.3) | <0.01 |
| Sex, n (%) |  |  |  | <0.01 |
| Female | 1666 (60.8) | 767 (69.0) | 899 (55.3) |  |
| Male | 1072 (39.2) | 344 (31.0) | 728 (44.7) |  |
| Education, n (%) |  |  |  | <0.01 |
| Below high school | 771 (30.9) | 417 (41.7) | 354 (23.7) |  |
| High school | 1256 (50.4) | 450 (45.0) | 806 (53.9) |  |
| College or above | 467 (18.7) | 133 (13.3) | 334 (22.4) |  |
| Marital status, n (%) |  |  |  | <0.01 |
| Married or partnered | 1938 (70.8) | 709 (63.8) | 1229 (75.5) |  |
| Other marital status | 800 (29.2) | 402 (36.2) | 398 (24.5) |  |
| Drinking status, n (%) |  |  |  | <0.01 |
| Never drinkers | 171 (6.6) | 104 (10.2) | 67 (4.3) |  |
| Ever drinkers | 2401 (93.4) | 911 (89.8) | 1490 (95.7) |  |
| Smoking status, n (%) |  |  |  | 0.03 |
| Never smokers | 1140 (41.7) | 435 (39.2) | 705 (43.4) |  |
| Ever smokers | 1595 (58.3) | 675 (60.8) | 920 (56.6) |  |
| SBP, mean (SD), mmHg | 132.5 (17.6) | 133.0 (18.0) | 132.1 (17.3) | 0.24 |
| DBP, mean (SD), mmHg | 75.7 (10.4) | 75.4 (10.7) | 75.9 (10.3) | 0.2 |
| Grip strength, mean (SD), kg | 32.1 (11.2) | 29.3 (10.6) | 34.0 (11.2) | <0.01 |

**Supplementary Table 4. Baseline Characteristics of the HRS Population**

| **Variables** | **Overall** | **Injury Group** | **Non-injury Group** | ***p*** |
| --- | --- | --- | --- | --- |
| Number | 4817 | 2744 | 2073 |  |
| Age, mean (SD), years | 74.0 (6.9) | 74.5 (7.1) | 73.4 (6.5) | <0.01 |
| Sex, n (%) |  |  |  | <0.01 |
| Female | 2944 (61.1) | 1821 (66.4) | 1123 (54.2) |  |
| Male | 1873 (38.9) | 923 (33.6) | 950 (45.8) |  |
| Education, n (%) |  |  |  | <0.01 |
| Below high school | 878 (18.2) | 606 (22.1) | 272 (13.1) |  |
| High school | 2798 (58.1) | 1608 (58.6) | 1190 (57.4) |  |
| College or above | 1139 (23.7) | 529 (19.3) | 610 (29.4) |  |
| Marital status, n (%) |  |  |  | <0.01 |
| Married or partnered | 2869 (59.6) | 1528 (55.7) | 1341 (64.7) |  |
| Other marital status | 1948 (40.4) | 1216 (44.3) | 732 (35.3) |  |
| Drinking status, n (%) |  |  |  | <0.01 |
| Never drinkers | 2142 (44.5) | 1331 (48.5) | 811 (39.1) |  |
| Ever drinkers | 2675 (55.5) | 1413 (51.5) | 1262 (60.9) |  |
| Smoking status, n (%) |  |  |  | 0.52 |
| Never smokers | 2164 (45.3) | 1220 (44.9) | 944 (45.8) |  |
| Ever smokers | 2616 (54.7) | 1500 (55.1) | 1116 (54.2) |  |
| SBP, mean (SD), mmHg | 132.9 (20.4) | 132.0 (20.3) | 134.2 (20.5) | 0.02 |
| DBP, mean (SD), mmHg | 78.5 (11.1) | 77.9 (11.4) | 79.3 (10.7) | 0.01 |
| Grip strength, mean (SD), kg | 30.0 (10.5) | 28.3 (10.0) | 32.2 (10.8) | <0.01 |

**Supplementary Table 5. Sensitivity analysis I: Association between Baseline IC and CMM**

|  | CHARLS | | ELSA | | HRS | | Pooled | |
| --- | --- | --- | --- | --- | --- | --- | --- | --- |
|  | HR (95% CI) | *P* | HR (95% CI) | *P* | HR (95% CI) | *P* | HR (95% CI) | *P* |
| Model1 | 1.31 (1.15-1.48) | <0.001 | 1.69 (1.41-2.03) | <0.001 | 1.35 (1.20-1.52) | <0.001 | 1.51 (1.39-1.63) | <0.001 |
| Model2 | 1.26 (1.10-1.43) | <0.001 | 1.50 (1.23-1.83) | <0.001 | 1.34 (1.19-1.52) | <0.001 | 1.37 (1.27-1.49) | <0.001 |
| Model3 | 1.33 (1.16-1.52) | <0.001 | 1.41 (1.14-1.74) | 0.001 | 1.24 (1.03-1.49) | 0.027 | 1.35 (1.23-1.49) | <0.001 |

CHARLS China Health and Retirement Longitudinal Study, ELSA English Longitudinal Study of Ageing, HRS Health and Retirement Study. Model 1 was the unadjusted model (no covariates adjusted). Model 2 was adjusted for age, sex, education level, and marital status. Model 3 was further adjusted for smoking status, drinking status, SBP, DBP, and grip strength, based on Model 2.

**Supplementary Table 6. Sensitivity analysis Ⅱ: Association between Cumulative IC Scores and CMM**

|  | CHARLS | | ELSA | | HRS | | Pooled | |
| --- | --- | --- | --- | --- | --- | --- | --- | --- |
|  | HR (95% CI) | *P* | HR (95% CI) | *P* | HR (95% CI) | *P* | HR (95% CI) | *P* |
| T1 | 1 Ref. |  | 1 Ref. |  | 1 Ref. |  | 1 Ref. |  |
| T2 | 1.34 (1.13-1.60) | 0.001 | 1.37 (1.08-1.73) | 0.008 | 1.07 (0.83-1.39) | 0.579 | 1.26 (1.12-1.43) | <0.001 |
| T3 | 1.61 (1.37-1.89) | <0.001 | 2.15 (1.73-2.67) | <0.001 | 1.30 (1.05-1.61) | 0.016 | 1.59 (1.42-1.79) | <0.001 |

The cumulative IC scores are calculated by summing the IC score at baseline and that at the second survey. On this basis, three grades are classified:T1 indicates the cumulative IC scores are intact;T2 indicates the cumulative IC scores are 1 standard deviation below the intact level;T3 indicates the cumulative IC scores are 1 standard deviation below the intact level more than 1 standard deviation below the intact level.

CHARLS China Health and Retirement Longitudinal Study, ELSA English Longitudinal Study of Ageing, HRS Health and Retirement Study.

All models were adjusted for age, sex, education level, marital status, smoking status, drinking status, SBP, DBP, and grip strength.

**Supplementary Table 7. Sensitivity analysis Ⅲ: Association between Changes in IC and CMM**

|  | CHARLS | | ELSA | | HRS | | Pooled | |
| --- | --- | --- | --- | --- | --- | --- | --- | --- |
|  | HR (95% CI) | *P* | HR (95% CI) | *P* | HR (95% CI) | *P* | HR (95% CI) | *P* |
| No decline | 1 Ref. |  | 1 Ref. |  | 1 Ref. |  | 1 Ref. |  |
| Decline | 1.43 (1.19-1.74) | <0.001 | 1.45 (1.09-1.93) | 0.010 | 1.06 (0.77-1.46) | 0.713 | 1.34 (1.16-1.54) | <0.001 |
| Improvement | 1.29 (1.05-1.57) | 0.015 | 1.37 (1.05-1.80) | 0.022 | 1.22 (0.92-1.62) | 0.173 | 1.27 (1.10-1.46) | 0.001 |
| Persistent impairment | 1.65 (1.40-1.95) | <0.001 | 2.24 (1.79-2.80) | <0.001 | 1.27 (1.02-1.59) | 0.032 | 1.61 (1.43-1.81) | <0.001 |

The changes in IC is calculated by subtracting the IC score at baseline from that at the second survey. On this basis, four grades are classified,No decline: The IC is intact both at baseline and the second survey;Decline: The IC is intact at baseline but impaired at the second survey; Improvement: The IC is impaired at baseline but intact at the second survey; Persistent impairment: The IC is impaired both at baseline and the second survey.

CHARLS China Health and Retirement Longitudinal Study, ELSA English Longitudinal Study of Ageing, HRS Health and Retirement Study. All models were adjusted for age, sex, education level, marital status, smoking status, drinking status, SBP, DBP, and grip strength.

**Supplementary Table 8. Sensitivity analysis Ⅳ: Association between Baseline IC and CMM**

|  | CHARLS | | ELSA | | HRS | | Pooled | |
| --- | --- | --- | --- | --- | --- | --- | --- | --- |
|  | HR (95% CI) | *P* | HR (95% CI) | *P* | HR (95% CI) | *P* | HR (95% CI) | *P* |
| Model1 | 1.35 (1.17-1.56) | <0.001 | 1.31 (1.04-1.66) | 0.023 | 1.24 (1.01-1.52) | 0.039 | 1.38 (1.25-1.54) | <0.001 |
| Model2 | 1.34 (1.16-1.55) | <0.001 | 1.30 (1.02-1.64) | 0.031 | 1.34 (1.19-1.52) | <0.001 | 1.39 (1.25-1.54) | <0.001 |
| Model3 | 1.34 (1.16-1.55) | <0.001 | 1.28 (1.02-1.63) | 0.037 | 1.24 (1.02-1.53) | 0.035 | 1.41 (1.27-1.56) | <0.001 |

CHARLS China Health and Retirement Longitudinal Study, ELSA English Longitudinal Study of Ageing, HRS Health and Retirement Study. Model 1 was the unadjusted model (no covariates adjusted). Model 2 was adjusted for age, sex, education level, and marital status. Model 3 was further adjusted for smoking status, drinking status, SBP, DBP, and grip strength, based on Model 2.

**Supplementary Table 9. Sensitivity analysis Ⅴ: Association between Cumulative IC Scores and CMM**

|  | CHARLS | | ELSA | | HRS | | Pooled | |
| --- | --- | --- | --- | --- | --- | --- | --- | --- |
|  | HR (95% CI) | *P* | HR (95% CI) | *P* | HR (95% CI) | *P* | HR (95% CI) | *P* |
| T1 | 1 Ref. |  | 1 Ref. |  | 1 Ref. |  | 1 Ref. |  |
| T2 | 1.48 (1.22-1.80) | 0.001 | 1.25 (0.93-1.67) | 0.143 | 1.17 (0.89-1.53) | 0.272 | 1.32 (1.15-1.52) | <0.001 |
| T3 | 1.71 (1.43-2.05) | <0.001 | 1.73 (1.30-2.30) | <0.001 | 1.31 (1.04-1.67) | 0.023 | 1.66 (1.46-1.88) | <0.001 |

The cumulative IC scores are calculated by summing the IC score at baseline and that at the second survey. On this basis, three grades are classified:T1 indicates the cumulative IC scores are intact;T2 indicates the cumulative IC scores are 1 standard deviation below the intact level;T3 indicates the cumulative IC scores are 1 standard deviation below the intact level more than 1 standard deviation below the intact level.

CHARLS China Health and Retirement Longitudinal Study, ELSA English Longitudinal Study of Ageing, HRS Health and Retirement Study.

All models were adjusted for age, sex, education level, marital status, smoking status, drinking status, SBP, DBP, and grip strength.

**Supplementary Table 10. Sensitivity analysis Ⅵ: Association between Changes in IC and CMM**

|  | CHARLS | | ELSA | | HRS | | Pooled | |
| --- | --- | --- | --- | --- | --- | --- | --- | --- |
|  | HR (95% CI) | *P* | HR (95% CI) | *P* | HR (95% CI) | *P* | HR (95% CI) | *P* |
| No decline | 1 Ref. |  | 1 Ref. |  | 1 Ref. |  | 1 Ref. |  |
| Decline | 1.64 (1.31-2.04) | <0.001 | 1.47 (1.00-2.16) | 0.046 | 1.11 (0.80-1.54) | 0.529 | 1.37 (1.16-1.61) | <0.001 |
| Improvement | 1.37 (1.10-1.71) | 0.005 | 1.16 (0.84-1.61) | 0.365 | 1.23 (0.97-1.82) | 0.080 | 1.36 (1.16-1.59) | 0.001 |
| Persistent impairment | 1.76 (1.46-2.11) | <0.001 | 1.78 (1.33-2.39) | <0.001 | 1.29 (1.01-1.65) | 0.039 | 1.67 (1.47-1.91) | <0.001 |

The changes in IC is calculated by subtracting the IC score at baseline from that at the second survey. On this basis, four grades are classified,No decline: The IC is intact both at baseline and the second survey;Decline: The IC is intact at baseline but impaired at the second survey; Improvement: The IC is impaired at baseline but intact at the second survey; Persistent impairment: The IC is impaired both at baseline and the second survey.

CHARLS China Health and Retirement Longitudinal Study, ELSA English Longitudinal Study of Ageing, HRS Health and Retirement Study. All models were adjusted for age, sex, education level, marital status, smoking status, drinking status, SBP, DBP, and grip strength.

**Supplementary Table 11. Sensitivity analysis Ⅶ: The association between Baseline IC and CMM assessed when death is considered as a competitive risk factor**

|  | CHARLS | | ELSA | | HRS | | Pooled | |
| --- | --- | --- | --- | --- | --- | --- | --- | --- |
|  | HR (95% CI) | *P* | HR (95% CI) | *P* | HR (95% CI) | *P* | HR (95% CI) | *P* |
| Model1 | 1.25 (1.10-1.41) | <0.001 | 1.63 (1.37-1.94) | <0.001 | 1.23 (1.10-1.38) | <0.001 | 1.36 (1.26-1.47) | <0.001 |
| Model2 | 1.20 (1.06-1.36) | 0.003 | 1.43 (1.18-1.74) | <0.001 | 1.23 (1.10-1.38) | <0.001 | 1.27 (1.18-1.38) | <0.001 |
| Model3 | 1.26 (1.12-1.44) | <0.001 | 1.36 (1.10-1.67) | 0.004 | 1.18 (0.98-1.41) | 0.075 | 1.28 (1.17-1.41) | <0.001 |

CHARLS China Health and Retirement Longitudinal Study, ELSA English Longitudinal Study of Ageing, HRS Health and Retirement Study. Model 1 was the unadjusted model (no covariates adjusted). Model 2 was adjusted for age, sex, education level, and marital status. Model 3 was further adjusted for smoking status, drinking status, SBP, DBP, and grip strength, based on Model 2.

**Supplementary Table 12. Sensitivity analysis Ⅷ: The association between Cumulative IC Scores and CMM assessed when death is considered as a competitive risk factor**

|  | CHARLS | | ELSA | | HRS | | Pooled | |
| --- | --- | --- | --- | --- | --- | --- | --- | --- |
|  | HR (95% CI) | *P* | HR (95% CI) | *P* | HR (95% CI) | *P* | HR (95% CI) | *P* |
| T1 | 1 Ref. |  | 1 Ref. |  | 1 Ref. |  | 1 Ref. |  |
| T2 | 1.31 (1.11-1.54) | 0.001 | 1.20 (0.93-1.55) | 0.150 | 1.07 (0.84-1.36) | 0.600 | 1.47 (1.32-1.65) | <0.001 |
| T3 | 1.52 (1.31-1.76) | <0.001 | 1.62 (1.26-2.09) | 0.001 | 1.18 (0.96-1.45) | 0.110 | 1.25 (1.11-1.40) | <0.001 |

The cumulative IC scores are calculated by summing the IC score at baseline and that at the second survey. On this basis, three grades are classified:T1 indicates the cumulative IC scores are intact;T2 indicates the cumulative IC scores are 1 standard deviation below the intact level;T3 indicates the cumulative IC scores are 1 standard deviation below the intact level more than 1 standard deviation below the intact level.

CHARLS China Health and Retirement Longitudinal Study, ELSA English Longitudinal Study of Ageing, HRS Health and Retirement Study.

All models were adjusted for age, sex, education level, marital status, smoking status, drinking status, SBP, DBP, and grip strength.

**Supplementary Table 13. Sensitivity analysis Ⅸ: The association between Changes in IC and CMM assessed when death is considered as a competitive risk factor**

|  | CHARLS | | ELSA | | HRS | | Pooled | |
| --- | --- | --- | --- | --- | --- | --- | --- | --- |
|  | HR (95% CI) | *P* | HR (95% CI) | *P* | HR (95% CI) | *P* | HR (95% CI) | *P* |
| No decline | 1 Ref. |  | 1 Ref. |  | 1 Ref. |  | 1 Ref. |  |
| Decline | 1.41 (1.18-1.68) | <0.001 | 1.24 (0.91-1.68) | 0.17 | 0.99 (0.74-1.35) | 0.980 | 1.49 (1.33-1.67) | <0.001 |
| Improvement | 1.23 (1.01-1.49) | 0.037 | 1.18 (0.88-1.58) | 0.27 | 1.23 (0.94-1.61) | 0.140 | 1.30 (1.13-1.49) | <0.001 |
| Persistent impairment | 1.56 (1.33-1.82) | <0.001 | 1.69 (1.30-2.19) | <0.001 | 1.16 (0.94-1.42) | 0.170 | 1.24 (1.08-1.43) | 0.002 |

The changes in IC is calculated by subtracting the IC score at baseline from that at the second survey. On this basis, four grades are classified,No decline: The IC is intact both at baseline and the second survey;Decline: The IC is intact at baseline but impaired at the second survey; Improvement: The IC is impaired at baseline but intact at the second survey; Persistent impairment: The IC is impaired both at baseline and the second survey.

CHARLS China Health and Retirement Longitudinal Study, ELSA English Longitudinal Study of Ageing, HRS Health and Retirement Study. All models were adjusted for age, sex, education level, marital status, smoking status, drinking status, SBP, DBP, and grip strength.

**Supplementary Table 14. Sensitivity analysis Ⅹ: Association of Five Categories of Change in IC with Incident CMM**

|  | CHARLS | | ELSA | | HRS | | Pooled | |
| --- | --- | --- | --- | --- | --- | --- | --- | --- |
|  | HR (95% CI) | *P* | HR (95% CI) | *P* | HR (95% CI) | *P* | HR (95% CI) | *P* |
| No decline | 1 Ref. |  | 1 Ref. |  | 1 Ref. |  | 1 Ref. |  |
| Decline | 1.43 (1.19-1.74) | <0.001 | 1.45 (1.09-1.93) | 0.010 | 1.06 (0.77-1.46) | 0.713 | 1.34 (1.16-1.54) | <0.001 |
| Minor improvement | 1.31 (1.05-1.64) | 0.018 | 1.39 (1.05-1.84) | 0.021 | 1.13 (0.83-1.53) | 0.436 | 1.26 (1.08-1.47) | 0.003 |
| Major improvement | 1.21 (0.83-1.76) | 0.316 | 1.22 (0.57-2.61) | 0.602 | 1.81 (1.05-3.11) | 0.031 | 1.29 (0.96-1.72) | 0.089 |
| Persistent impairment | 1.65 (1.40-1.95) | <0.001 | 2.24 (1.79-2.80) | <0.001 | 1.27 (1.02-1.59) | 0.032 | 1.61 (1.43-1.81) | <0.001 |

The changes in IC is calculated by subtracting the IC score at baseline from that at the second survey. No decline (The IC is intact both at baseline and the second survey), Decline (The IC is intact at baseline but impaired at the second survey), Minor improvement (Baseline IC shows mild impairment (IC = 5), and becomes intact at the second survey), Major improvement (Baseline IC shows substantial impairment (IC < 5), and becomes intact at the second survey), Persistent impairment (The IC is impaired both at baseline and the second survey).

CHARLS China Health and Retirement Longitudinal Study, ELSA English Longitudinal Study of Ageing, HRS Health and Retirement Study. All models were adjusted for age, sex, education level, marital status, smoking status, drinking status, SBP, DBP, and grip strength.

**Supplementary Table 15. Sensitivity analysis Ⅺ: Pooled associations between baseline IC and incident CMM across single-disease subgroups**

| Pooled | | | | | | | | |
| --- | --- | --- | --- | --- | --- | --- | --- | --- |
|  | hypertension | | diabetes | | heart disease | | stroke | |
|  | HR (95% CI) | *P* | HR (95% CI) | *P* | HR (95% CI) | *P* | HR (95% CI) | *P* |
| Model1 | 1.39 (1.24-1.56) | <0.001 | 1.49 (1.07-2.07) | 0.019 | 1.35 (1.07-1.70) | 0.011 | 1.22 (0.59-2.55) | 0.591 |
| Model2 | 1.35 (1.20-1.52) | <0.001 | 1.35 (0.96-1.89) | 0.081 | 1.27 (1.00-1.62) | 0.047 | 1.56 (0.69-3.54) | 0.286 |
| Model3 | 1.25 (1.08-1.45) | 0.003 | 1.24 (0.81-1.90) | 0.313 | 1.31 (0.99-1.75) | 0.058 | 0.69 (0.23-2.14) | 0.524 |

CHARLS China Health and Retirement Longitudinal Study, ELSA English Longitudinal Study of Ageing, HRS Health and Retirement Study. Model 1 was the unadjusted model (no covariates adjusted). Model 2 was adjusted for age, sex, education level, and marital status. Model 3 was further adjusted for smoking status, drinking status, SBP, DBP, and grip strength, based on Model 2.

**Supplementary Table 16. Sensitivity analysis Ⅻ: Pooled associations between cumulative IC scores and incident CMM across single-disease subgroups**

| Pooled | | | | | | | | |
| --- | --- | --- | --- | --- | --- | --- | --- | --- |
|  | hypertension | | diabetes | | heart disease | | stroke | |
|  | HR (95% CI) | *P* | HR (95% CI) | *P* | HR (95% CI) | *P* | HR (95% CI) | *P* |
| T1 | 1 Ref. |  | 1 Ref. |  | 1 Ref. |  | 1 Ref. |  |
| T2 | 1.11 (0.92-1.35) | 0.269 | 1.13 (0.65-1.94) | 0.672 | 0.94 (0.63-1.40) | 0.762 | 6.85 (1.15-40.48) | 0.033 |
| T3 | 1.45 (1.22-1.72) | <0.001 | 1.61 (0.97-2.67) | <0.060 | 1.49 (1.06-2.10) | <0.020 | 2.07 (0.36-12.09) | 0.418 |

The cumulative IC scores are calculated by summing the IC score at baseline and that at the second survey. On this basis, three grades are classified:T1 indicates the cumulative IC scores are intact;T2 indicates the cumulative IC scores are 1 standard deviation below the intact level;T3 indicates the cumulative IC scores are 1 standard deviation below the intact level more than 1 standard deviation below the intact level.

CHARLS China Health and Retirement Longitudinal Study, ELSA English Longitudinal Study of Ageing, HRS Health and Retirement Study.

All models were adjusted for age, sex, education level, marital status, smoking status, drinking status, SBP, DBP, and grip strength.

**Supplementary Table 17. Sensitivity analysis ⅩⅢ: Pooled associations between changes in IC and incident CMM across single-disease subgroups**

| Pooled | | | | | | | | |
| --- | --- | --- | --- | --- | --- | --- | --- | --- |
|  | hypertension | | diabetes | | heart disease | | stroke | |
|  | HR (95% CI) | *P* | HR (95% CI) | *P* | HR (95% CI) | *P* | HR (95% CI) | *P* |
| No decline | 1 Ref. |  | 1 Ref. |  | 1 Ref. |  | 1 Ref. |  |
| Decline | 1.21 (0.97-1.51) | 0.094 | 1.36 (0.75-2.41) | 0.318 | 1.04 (0.63-1.70) | 0.884 | 7.53 (1.25-45.37) | 0.027 |
| Improvement | 1.09 (0.87-1.36) | 0.442 | 1.20 (0.61-2.35) | 0.592 | 0.92 (0.59-1.44) | 0.716 | 7.23 (0.88-58.95) | 0.064 |
| Persistent impairment | 1.47 (1.23-1.75) | <0.001 | 2.50 (0.88-2.52) | 0.136 | 1.57 (1.11-2.22) | 0.011 | 1.74 (0.28-10.67) | 0.550 |

The changes in IC is calculated by subtracting the IC score at baseline from that at the second survey. On this basis, four grades are classified,No decline: The IC is intact both at baseline and the second survey;Decline: The IC is intact at baseline but impaired at the second survey; Improvement: The IC is impaired at baseline but intact at the second survey; Persistent impairment: The IC is impaired both at baseline and the second survey.

CHARLS China Health and Retirement Longitudinal Study, ELSA English Longitudinal Study of Ageing, HRS Health and Retirement Study. All models were adjusted for age, sex, education level, marital status, smoking status, drinking status, SBP, DBP, and grip strength.
